# Education Research: Shaping the Future of Epilepsy Fellowship Training in Canada: A Canadian League Against Epilepsy Delphi Study

**DOI:** 10.1212/NE9.0000000000200287

**Published:** 2026-01-15

**Authors:** Anita N. Datta, Kristin M. Ikeda, Carlos Ivan Salazar Cerda, Aristides Hadjinicolaou, Gianluca D'Onofrio, Julia Jacobs-LeVan, Juan Pablo Appendino, Eliane Kobayashi, Rajesh Ramachandrannair, Esther Bui

**Affiliations:** 1 Division of Neurology, BC Children's Hospital, University of British Columbia, Vancouver, Canada;; 2 Division of Neurology, The Queen Elizabeth II Health Sciences Centre, Dalhousie University, Halifax, Nova Scotia, Canada;; 3 Division of Neurology, Hospital for Sick Children, University of Toronto, Canada;; 4 Division of Neurology, CHU Sainte-Justine, Université de Montréal, Quebec, Canada;; 5 Division of Neurology, Centre Hospitalier de l'Université de Montréal, Quebec, Canada;; 6 Division of Neurology, Alberta Children's Hospital, University of Calgary, Alberta, Canada;; 7 Division of Neurology. Jim Pattison Children's Hospital. University of Saskatchewan. Saskatoon, Canada;; 8 Department of Neurology and Neurosurgery, Montreal Neurological Institute, McGill University, Quebec, Canada;; 9 Department of Neurology, Peter O'Donnell Brain Institute, University of Texas Southwestern Medical Center, Dallas;; 10 Division of Neurology, McMaster Children's Hospital, McMaster University, Hamilton, Ontario, Canada; and; 11 Division of Neurology, Toronto Wester Hospital, University of Toronto, Ontario, Canada.

## Abstract

**Background and Objectives:**

Epileptologists are neurologists with advanced training in the diagnosis and management of complex epilepsy. In Canada, epilepsy fellowship programs vary considerably in duration and content because of the lack of formal accreditation or standardized curricula. Contributing factors include regional differences in resources, infrastructure, and program-specific goals.

**Methods:**

To address this variability, the Canadian League Against Epilepsy established a working group to develop a national consensus framework for postgraduate epilepsy education. The process started with a comprehensive literature review of North American and international curricula and standards to ensure alignment. The nominal group technique involved 4 rounds with Canadian epilepsy educators, focusing on idea generation, sharing, discussion, and questionnaire completion on key topics. A 70% agreement threshold was set to establish consensus. Items not reaching this threshold were revisited in a final discussion round. Recorded discussions were analyzed, with the eventual goal of incorporating these findings into a national consensus guideline for epilepsy fellowship training in Canada.

**Results:**

Agreement was reached on core topics such as eligibility, fellowship duration, learning objectives, timing of skill acquisition, epilepsy surgery exposure, research involvement, evaluation methods, and accreditation. Areas lacking consensus underwent further exploration in a final survey round. The process also highlighted priorities for future work, including program accreditation and the development of national clinical examinations.

**Discussion:**

This study represents a foundational step in establishing unified standards for epilepsy fellowship training in Canada and reinforces the importance of national collaboration to promote excellence in epilepsy and EEG education.

## Introduction

Epilepsy is a neurologic disorder characterized by a predisposition to seizures.^1^ It affects approximately 1% of the population, with approximately one-third of individuals experiencing drug-resistant epilepsy requiring specialized care and advanced treatments.^2,3^ An epileptologist is a neurologist with advanced training in diagnosing and managing complex epilepsy. They have expertise in areas such as electroencephalography (EEG), neuroimaging, presurgical evaluation, neuromodulation, and dietary therapies.

In Canada, while standardized education programs are available in certain neurologic specialties, such as neuromuscular disorders,^4^ there is a pressing need for accredited standardized epilepsy fellowship programs and a unified curriculum for epilepsy fellowship programs. The only certification currently available is the EEG examination administered by the Canadian Society of Clinical Neurophysiology (CSCN).^5^ In the United States, the American Board of Neurology and Psychiatry recognized clinical neurophysiology (1990)^6^ and epilepsy (2010)^7^ as subspecialties in neurology with defined learning objectives and certification examinations. The Accreditation Council for Graduate Medical Education (ACGME) has since approved standardized fellowships in these areas.^8,9^ Other efforts for standardized education include the American Board of Clinical Neurophysiology's certification examination,^10^ the American Epilepsy Society's curriculum for fellows,^11^ and the International League Against Epilepsy's virtual academy.^12^

In Canada, the content and duration of fellowships are variable, ranging from 6 months to 3 years. Heterogeneity by geographic location, available resources, and infrastructure can result in program-specific goals and objectives. When comparing our vision of a Canadian epilepsy fellowship with that in the United States and other countries, several distinguishing features emerge. Notably, unlike other jurisdictions, Canada currently lacks a formal accreditation structure, which is linked to funding and standardized evaluations. Furthermore, we have a unique health care system, diverse geography, and interprovincial health care differences. Although this may initially seem as a limitation, it presents a valuable opportunity for us to develop a framework that is specifically tailored to Canadian needs. The core objective of this endeavor is to develop a national framework to train epileptologists who can provide exceptional, evidence-based care by effectively using available resources. While obtaining guidance from international models, we focus on creating a uniquely Canadian framework that aligns with our health care values and addresses the diverse neurologic needs of the population.

The Canadian League Against Epilepsy (CLAE), a national organization comprising educators, advocates, health care professionals, and researchers, shares a common goal of improving the lives of people with epilepsy. In the context of these challenges, a working group of the CLAE aimed to establish a consensus-based standardized content and organization for a curriculum for epilepsy postgraduate education among national postgraduate epilepsy educators using a Delphi technique. This technique is based on the premise that expert consensus enhances individual judgment and the collective experience can inform suitable alternatives for designing these programs.^13^ The objective of this article was to describe the process of developing, for the first time in Canada, a consensus-based standardized content and organization for a curriculum for postgraduate epilepsy education. By providing a detailed outline of this developmental process, we aim to inspire other postgraduate curriculum development initiatives across medical education.

## Methods

In October 2021, the CLAE established a postgraduate epilepsy education core working group consisting of 3 epileptologists (2 adults, 1 pediatric) and 2 epilepsy fellows (1 adult, 1 pediatric). A nominal group and Delphi technique was used to develop a national framework for postgraduate epilepsy education in Canada. No consulting about the method took place.

The consensus development began with a comprehensive literature review to assess current North American and international standards in epilepsy education, focusing on aims, content, learning outcomes, teaching methods, and evaluation techniques. Searches were conducted using MEDLINE, PubMed, and Embase, along with a review of educational institution websites. The reviewed documents included key criteria for epilepsy fellowships, such as those established by the ACGME,^8,9^ as well as data from board examinations, including the American Board of Neurology and Psychiatry subspecialties of clinical neurophysiology^6^ and epilepsy^7^ and the American Board of Clinical Neurophysiology's certification examination.^10^ In addition, educational curricula from organizations such as the American Epilepsy Society^11^ and courses provided by the International League Against Epilepsy were reviewed.^12^

An expert panel was convened, consisting of Canadian epilepsy fellowship program directors, experienced clinical directors, and 3 leaders from the Canadian Epilepsy Teaching Network at the CLAE, who were informed about the Delphi study and invited to participate. The panel included a total of 22 members, with sociodemographic and practice-related details provided in Table 1. The goal was that by incorporating analyses of current standard frameworks alongside expert opinion would provide a more comprehensive understanding of best practices in Canada and ensure that recommendations are grounded in both empirical evidence and established benchmarks.

**Table 1:** Demographics of the Expert Panel

Sex	N = 22 (%)
Male	11 (50)
Age	
25–34	1 (5)
35–44	12 (55)
45–54	6 (27)
55–65	3 (14)
Years of practice	
0–5	3 (14)
6–10	9 (41)
11–15	5 (23)
15–20	2 (10)
>20	3 (14)
Location^a^	
Western	7 (32)
Central	13 (59)
Atlantic	2 (10)
Academic position^b^	
Clinician teacher	13 (59)
Clinician educator	12 (55)
Fellowship director	11(50)
Clinician scientist	5 (23)
Administrator^c^	5 (23)
Residency program director	1 (5)
Other	1 (5)
Fellowship positions	
0	4 (18)
1	8 (36)
2	7 (32)
3	1 (5)
4	1 (5)
5	1 (5)
Funded positions	N = 13
0	4
1	8–9^d^
2	2–5^d^
3	0–1^d^

Central Canada refers to Ontario and Quebec.

Atlantic Canada refers to Newfoundland and Labrador, Nova Scotia, Prince Edward Island, and New Brunswick.

a Western Canada refers to British Columbia, Alberta, Saskatchewan, and Manitoba.

b Some individuals have more than 1 type of academic position.

c Administrator is a position that involves overseeing the nonteaching or clinical aspects of an education program.

d A range exists due to variable funding availability.

**Figure 1:**
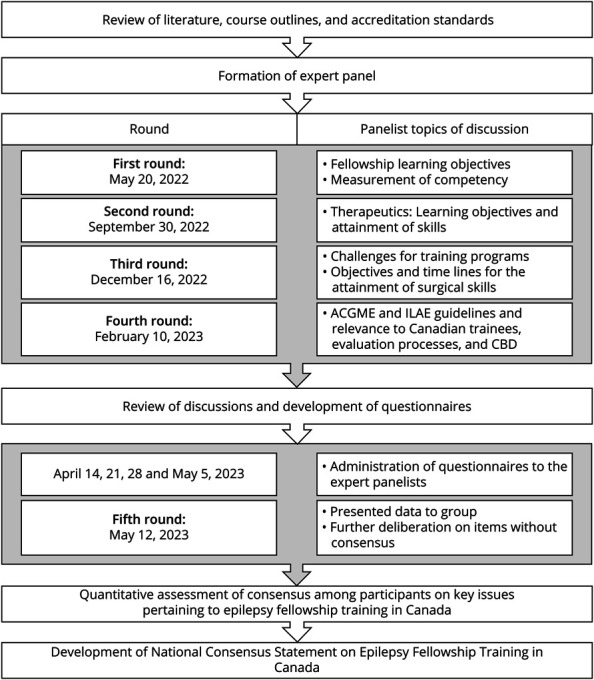
Time Line of Study Stages ACGME = Accreditation Council for Graduate Medical Education; CBME = competency-based medical education; ILAE = International League Against Epilepsy.

The rounds of the Delphi process are shown in Figure 1. Panelists were required to attend a minimum of 4 of the 5 rounds and complete a deidentified survey of their practice characteristics. Initial deliberations used a nominal group technique designed to replicate decision-making processes in curriculum development. The nominal group technique is a structured group decision-making method designed to facilitate equal participation and generate ideas or reach consensus among participants. Serial meetings were held where all expert panel members could contribute their expertise equally. The method was particularly valuable here because it allowed the diverse group of Canadian epilepsy experts to systematically identify and prioritize the most important elements for postgraduate epilepsy training, drawing from their collective experience while maintaining objectivity in the decision-making process. Four rounds of discussions gathered insights into various aspects of fellowship training and were guided by documents such as the Epilepsy Milestones from ACGME^14^ and the ILAE's competency-based curriculum.^15^

After the discussions, 4 questionnaires were created to gauge consensus on key topics and administered in a fifth and final round. The questionnaires are given in eAppendix 1. The first questionnaire covered training aspects, including eligibility, learning objectives, and evaluation methods. Based on the ILAE commission report, 3 subsequent questionnaires on learning objectives were created and divided as ILAE1—*Diagnostics*, ILAE2—*Therapeutics/Counseling*, and ILAE3—*Surgery/Emergencies/Biology of Epilepsy*.^15^ Panelists were asked to respond whether learning objectives should be acquired by (1) the end of residency, (2) the end of first-year fellowship, or (3) the end of a second-year fellowship. Delphi consensus was set at 70% agreement based on expert opinion and literature review of the median threshold accepted as consensus in previous studies using the Delphi technique for health care consensus.^16-18^ The expert panelists were shown the aggregated statistical results with mean values plus the summary of the open responses. Items without consensus were addressed in a final round, which included all the 22 expert panelists. All rounds were audio-recorded and transcribed verbatim.

### Standard Protocol Approvals, Registrations, and Participant Consents

This study was approved by the University of Toronto Ethics Board under the RIS Human Protocol 42597.

### Data Availability

Anonymized data not published within this article will be made available by request from qualified investigators.

## Results

The results provide the group's consensus on various topics, including fellowship eligibility, objectives, duration, and the comparison of time-based and competency-based learning. It also addresses accreditation, evaluation methods, and research. The quantitative items were descriptively analyzed. The responses to the 4 questionnaires and the achieved consensus are displayed in eFigures 1 to 4.

### Fellowship Eligibility

There was unanimous agreement that epilepsy fellowship trainees must be trained neurologists or possess a foreign equivalent, as trainees from other countries may not be required to have a neurology certification to practice as epileptologists in their home countries (Q1:1). Everyone concurred that each institution should implement a formalized teaching curriculum for fellows, which includes consistent training objectives for both Canadian and foreign trainees (Q1:20).

### Fellowship Duration

In Canada, several programs offer 6 months of training in EEG interpretation. Forty-two percent of panelists expressed support for six-month fellowships aimed at achieving competence in routine EEG interpretation or preparing for the CSCN examination preparation (Q1:22). However, there was a consensus that epilepsy fellowships should last at least 1 year.

### Learning Objectives and Timing of Skill Acquisition

There was complete consensus that standardized ILAE clinical and EEG training objectives must be met before completing the first or second year of fellowship (Q1:3). All panelists agreed that the ILAE roadmap learning objectives are relevant to Canadian trainees and that most skills should be achieved by the end of the first year, except for recognizing ictal and interictal patterns with intracranial recordings (ILAE3#26), which should be attained by the end of the second year. There was no consensus on when to acquire epilepsy surgery skills (ILAE1:27, ILAE3:2, and 6). Furthermore, all panelists agreed that a CLAE consensus statement should not include a minimum number of EEGs, continuous EEGs (cEEGs), or surgical cases that need to be completed in an epilepsy fellowship (Q1:2).

Regarding critical care skills, there was complete consensus that, by the end of the first year of fellowship, epilepsy fellows should be competent in identifying emergency conditions (ILAE 3:15) and managing or advising on prolonged or sequential seizures (ILAE 3:16). In addition, fellows should demonstrate proficiency in diagnosing status epilepticus in both children and adults (ILAE 3:20); managing all types of convulsive status epilepticus, including EEG monitoring of treatment effects (ILAE 3:21); and managing nonconvulsive status epilepticus with EEG guidance (ILAE 3:21).

#### Epilepsy Surgery Skills

Presurgical epilepsy evaluations are personalized and progress in complexity, including invasive procedures, thus requiring specialized training in an epilepsy fellowship. Eighty percent agreed that such evaluations should be mandatory in the first or second year of fellowship (Q1:19). All panelists agreed that, by the end of the first year, fellows should be competent in presenting and discussing surgical cases with a multidisciplinary team, though not necessarily making surgical decisions (Q1:24).

There was no consensus among panelists on when advanced surgical skills should be attained, including demonstrating working knowledge on advanced presurgical techniques (ILAE3:2), planning implantation of intracranial electrodes (ILAE3:6), and knowledge on electromagnetic source imaging (ILAE1:27). In the discussions, it was noted that lack of agreement may have been due to confusion over terminology, as working knowledge may imply different levels of involvement than actively participating in procedures.

Forty-four percent agreed that stereo-electroencephalography (sEEG) and invasive monitoring should be mandatory in the second year of a 2-year epilepsy fellowship (Q1:25). After clarifying that advanced skills involve active participation in surgical planning and interpretation, most panelists indicated that the timing of skill acquisition depends on case exposure at the institution. Trainees at low-volume centers should acquire these skills later, whereas those at high-volume centers may acquire them earlier.

All agreed that, by the end of year 2, fellows should have advanced knowledge of surgical management and planning (Q1:30). However, despite consensus on this learning objective, it was also understood that not all epilepsy fellowship programs in Canada perform epilepsy surgery or invasive EEG monitoring. Terminology, such as “mandatory,” could have negative repercussions for some programs. Another suggestion was for fellows to obtain advanced surgical skills at other centers, but this would require further meetings and collaboration, which stands beyond the scope of this project.

### Content Regarding Time-Based vs Competency-Based Design Models

The shift to learning outcomes has significantly changed how we assess competence for independent practice. The Royal College of Physicians and Surgeons of Canada (RCPSC) has implemented competency-based medical education (CBME) in residency evaluations, emphasizing workplace-based assessments through direct observation.^18^ Only 36% of panelists agreed that epilepsy fellowships should be based on CBME rather than a fixed time frame (Q1:9). Individual epilepsy fellowship programs often lack the infrastructure and funding to implement CBME and entrustable professional activities (EPAs) because they do not have a dedicated accreditation body to support such requirements. In Canada, most medical training programs are accredited by the Royal College of Physicians and Surgeons, which sets standards for competency-based education and provides platforms for EPA assessment. Without a formal accreditation, epilepsy fellowships are not integrated into these frameworks, and essential resources, such as EPA assessment platforms, often require additional funding. This lack of accreditation poses significant challenges to establishing standardized competency-based training in epilepsy fellowships.

### Accreditation

Some subspecialty areas of neurology, including neuromuscular medicine, are accredited and recognized by the RCPSC as areas of focused competency (AFC).^19^ Sixty percent agreed that epilepsy fellowships in Canada should be eventually formalized under the RCPSC (Q1:18). During the discussions, the panelists did not believe that accreditation of epilepsy programs by the RCPSC would be a feasible option for epilepsy fellowships at this time due to various factors, including infrastructure and personal requirements, time constraints, and substantial costs involved. This is a topic that would benefit from a dedicated subcommittee for further discussion.

### Examinations and Fellowship Evaluations

There are no standardized examinations for epilepsy fellows, apart from the CSCN, which provides a national EEG certification examination to assess competency in reading routine EEGs in Canada, excluding clinical epilepsy and invasive EEG studies. Not all provinces require the successful completion of this examination for EEG reading privileges. There was no complete consensus, as 64% agreed that the CSCN EEG examination should be mandatory for an epilepsy fellowship (Q1:13). Seventy-six percent of the panelists agreed that further work and collaboration with CSCN are required to establish a cEEG and epilepsy surgery examination for epilepsy fellows (Q1:14).

At present, there are no standardized ways that fellowship programs are evaluated or how fellows are evaluated, within a program. Ninety-six percent of the panelists agreed that epilepsy fellowship programs should undergo external reviews for quality assurance. Eighty-four percent of the panelists agreed that Canadian epilepsy centers should have standardized evaluation forms for fellows.

### Research

Only 32% of the panelists agreed that research should be mandatory in clinical epilepsy fellowship programs (Q1:7). In comparison, 54% suggested that it should be included in the second year (Q1:8). After further discussion, panelists concluded that research should not be mandatory, prioritizing clinical neurophysiology and epilepsy training, but some recommended it for the second year.

### Exposure to Pediatric and Adult Patients/Transition

Panelists emphasized the importance of adult fellows experiencing pediatric cases and vice versa, with 68% recommending mandatory exposure to pediatric-to-adult transition clinics (Q1:38). They also agreed that adult epilepsy fellows should undertake 1–3 months of pediatric rotations and pediatric fellows should do the same for adults (Q1:11). However, 36% suggested that cross-population exposure should be limited to EEG reading only. In comparison, another 36% advocated for including clinics, EEG, and EMU (Q1:12).

A summary of the consensus recommendations is provided in Table 2.

**Table 2:** Summary of Consensus Recommendations for Canadian Epilepsy Fellowship Training

Domain	Recommendation
Eligibility	• Completed neurology residency training or equivalent
Program duration	• Minimum 1 y
Program structure	• Formalized teaching curriculum with stated objectives • Standardized evaluation form for fellows • External review process for quality assurance
Learning framework	• ILAE road map learning objectives applicable to Canadian training programs • ILAE clinical and EEG training objectives represent minimal standards for 1-y fellowship completion
1-Year fellowship competencies	• Core ILAE clinical and EEG objectives (excluding intracranial EEG interpretation) • Emergency management of prolonged or sequential seizures • Diagnosis and management of convulsive and non-convulsive status epilepticus, including EEG monitoring of treatment response • Preparation, presentation, and discussion of surgical cases within multidisciplinary teams • National EEG certification examination (strongly encouraged)
2-Year fellowship competencies	• Advanced surgical management and planning • Intracranial EEG interpretation
Assessment methods	• Competency-based metrics and entrustable professional activities supported but limited at the present time by funding and infrastructure constraints
Research requirements	• Supported but not mandatory for 1-y clinical epilepsy fellowships

## Discussion

This article describes the methods used for the first attempt to establish a national consensus-based curriculum for epilepsy fellowship programs in Canada. The goal is to eventually publish a national consensus statement outlining a mission for epilepsy fellowship training in Canada. Currently, there is no gold standard for designing epilepsy fellowship curricula.^13^ The expert panelists who participated in the meetings provided practical working knowledge and diverse expertise in clinical epilepsy and education. The wide range of sociodemographic backgrounds of the panelists was a strength of the study, and the virtual format made it feasible to perform in different locations across the country. The panelists showed high levels of engagement, which could reflect their interest in the topic or the fact that they were aware of being study partners, with the results based on their responses. It was also addressed that incorporating the perspectives of respected epilepsy educators nationwide to reach a formal consensus is relevant for current and future trainees, ensuring that their training meets recognized national standards.

Canadian epilepsy training institutions vary in size and infrastructure, including epilepsy monitoring units, surgical programs, and advanced diagnostic tests. There are also differences in the number of trainees, staff involved in training, and expertise in areas such as epilepsy surgery and genetics. This variability affects training content and duration, leading panelists to conclude that a national consensus statement should be broad and generalizable to increase adoption while acknowledging that recommendations may be tailored to local resources and clinical needs. There was agreement on some 'mandatory' terms, such as the inclusion of certain core topics in the curriculum. However, concerns were raised that this could adversely affect programs lacking specific resources, such as advanced surgical facilities or genetic testing capabilities. These concerns highlight the need for a balance between setting national standards and accommodating local variations.

American epilepsy fellowship programs have full accreditation by the ACGME, which is linked to funding opportunities, and differences exist because of Canada's publicly funded system. European models, particularly those in countries with universal health care systems, offer similar parallels to the Canadian context. However, Canada's vast geography, diverse population demographics, and specific regulatory environment necessitate a distinctly tailored approach. Our framework draws on international best practices while addressing uniquely Canadian challenges, such as serving remote and Indigenous communities, navigating provincial health care variations, and maximizing the utility of limited specialized resources. While obtaining guidance from international models, we focused on creating a uniquely Canadian framework that aligns with our health care values and addresses the diverse neurologic needs of the population. Ultimately, our goal is to prepare specialists who can excel within Canada's health care environment while maintaining the highest standards of epilepsy care.

Several limitations should be considered when interpreting these findings. The results reflect only the opinions of the participating experts. The consensus's validity relied on the questionnaire's specific wording; varying definitions of consensus could result in different outcomes, as seen in the final nominal round when clarifying terminology helped achieve agreement, such as the “definition of advanced surgery skills.” Pilot testing of the questionnaires was not performed, which would have further enhanced their validity, and the lack of participant stratification by demographics, such as adult or pediatric expertise, may have limited insights. Another challenge was the inability to guarantee anonymity during group discussions, which might have made participants hesitate to express differing views. However, this aspect is a fundamental part of the Delphi technique, as seeing anonymous responses encourages reconsidering opinions. In addition, the patient population size and geographic distribution were not directly taken into account when discussing the criteria for establishing a supply of epilepsy experts. Despite the challenges associated with this method, the Delphi technique remains valuable for achieving consensus on previously unresolved issues.

Epilepsy poses a significant burden on individuals and society, making the training of Canadian epileptologists essential. Advances in clinical care demand that education and practice evolve in complexity, with educators playing a crucial and respected role in maintaining standardized training. Using the Delphi technique, we aimed to describe the process we undertook to establish a national consensus for epilepsy fellowship programs in Canada. This process identified areas of consensus and areas for future improvements, including program accreditation and clinical examinations. Our study underscores the importance of collaboration between educators and institutions to enhance epilepsy and EEG teaching with the best practices in medical education.

## Glossary

ACGME: Accreditation Council for Graduate Medical Education
CBME: competency-based medical education
CLAE: Canadian League Against Epilepsy
CSCN: Canadian Society of Clinical Neurophysiology
EPA: entrustable professional activity
RCPSC: Royal College of Physicians and Surgeons of Canada
